# Robot-Aided Systems for Improving the Assessment of Upper Limb Spasticity: A Systematic Review

**DOI:** 10.3390/s20185251

**Published:** 2020-09-14

**Authors:** Rubén de-la-Torre, Edwin Daniel Oña, Carlos Balaguer, Alberto Jardón

**Affiliations:** Department of Systems Engineering and Automation, University Carlos III of Madrid, Avda. de la Universidad 30, 28911 Leganés, Spain; 100280166@alumnos.uc3m.es (R.d.-l.-T.); eona@ing.uc3m.es (E.D.O.); balaguer@ing.uc3m.es (C.B.)

**Keywords:** upper limb, spasticity, cooperative robots, robot-assisted rehabilitation, assessment

## Abstract

Spasticity is a motor disorder that causes stiffness or tightness of the muscles and can interfere with normal movement, speech, and gait. Traditionally, the spasticity assessment is carried out by clinicians using standardized procedures for objective evaluation. However, these procedures are manually performed and, thereby, they could be influenced by the clinician’s subjectivity or expertise. The automation of such traditional methods for spasticity evaluation is an interesting and emerging field in neurorehabilitation. One of the most promising approaches is the use of robot-aided systems. In this paper, a systematic review of systems focused on the assessment of upper limb (UL) spasticity using robotic technology is presented. A systematic search and review of related articles in the literature were conducted. The chosen works were analyzed according to the morphology of devices, the data acquisition systems, the outcome generation method, and the focus of intervention (assessment and/or training). Finally, a series of guidelines and challenges that must be considered when designing and implementing fully-automated robot-aided systems for the assessment of UL spasticity are summarized.

## 1. Introduction

Over the past 40 years, the research in the upper motor neuron syndrome has focused on spasticity [1]. Lance defined spasticity as a motor disorder characterized by an increase in tonic stretch reflexes with exaggerated tendon jerks, resulting from hyper-excitability of the stretch reflex [2]. Most of the scenarios where we commonly find this type of syndrome are after a stroke, a spinal cord injury, or another neurological disorder affecting the central nervous system (CNS). Consequently, the phenomenon of spasticity is complex due to the heterogeneity of symptoms and the nature of motor control. The management of spasticity primarily involves two perspectives: ameliorating and assessing the degree of spasticity.

On one side, pharmacological and non-pharmacological approach therapies haven been employed as a treatment for reducing the spasticity effects [3]. Regarding pharmacological treatment, Baclofen is considered one of the first-line countermeasures, but Bont-A Toxin is the most common medication used to mitigate the effects of spasticity [4]. An injection reduces muscle tone up to 3 months and improves the upper limb capacities [5]. Non-pharmacological treatments mainly involve physical interventions aiming to minimize changes in the viscoelastic properties of connective tissue, muscles and joints, and changing patterns of spams [6]. Furthermore, recent studies have confirmed that the combined use of Bont-A with physical rehabilitation shortens the patient recovery time [7]; however, more clinical trials are needed to support the analysis considering previous studies and reviews [8,9].

One the other side, the understanding and diagnosis of spasticity have evolved exponentially in the last decades [10], and the assessment procedure has been attempted with a variety of methods. At the very beginning, some scales were created to evaluate the level of the disorder [11], such as the modified Ashworth scale (MAS), the Tardieu scale, the Spam severity scale, among others [10]. Currently, these scales are still the golden standard in clinical practice despite novel approaches having been proposed [12]. However, the existing scales are based on the perception of the clinician that evaluates the patient’s spasticity through their perception, experience, and training over the years.

This approach might not be appropriate because it establishes a subjective magnitude based on human impression. Not all clinicians have the same background and finally, the evaluation could depend on multiple imperceptible details [13]. Furthermore, this interpretation of the patient’s spasticity could extend or reduce the rehabilitation process and modify the movements or the specified therapy used in the sessions [14]. Consequently, an objective measurement of spasticity is required, and for that purpose, the development of robot-based systems can help clinicians to objectify the assessment of different components of the syndrome.

The aim of this article is to review and encompass the evolution of the robots used to measure the spasticity in the upper limb, discussing the characteristics of the mechanism that supports and assists the clinician diagnosis. The research incorporates and classifies studies focused on four main criteria: measuring spasticity, targeting upper limb, use of an exoskeleton or an end-effector device and inclusion of clinical trials. The remainder of this paper is as follows: Section 2 provides an overview of spasticity management and its fundamentals. Section 3 summarizes the results of the systematic literature review, presenting also the inclusion criteria and review limitations. Section 4 reviews and discuss the table content according to relevant aspects for UL assessment. Section 5 propose a perspective for future investigations. Finally, conclusions from this study are presented in Section 6.

## 2. Spasticity Management: Assessment and Treatments

A variety of sensorimotor and cognitive limitations can appear following an upper motor neuron (UMN) lesion. In the case of sensorimotor problems, they can be sorted in ‘positive’ and ‘negative’ features [15,16]. The positive features involve abnormal reflex responses, spasticity, spasms, clonus, and dis-synergic movement patterns. The negative features include muscle weakness, loss of dexterity, and fatigue. The combination of these positive and negative features leads to the loss of functionality and, consequently, the UMN syndrome must be understood as a complex picture where spasticity is only one component [17]. However, a particular focus on spasticity is considered under the premise that spasticity affects functional recovery and results in secondary complications like contractures, weakness, and pain.

In this way, spasticity can be considered globally as a disorder or disruption on voluntary control of muscles and stretch reflexes caused by damage in the central nervous system. The specific pathophysiology of spasticity remains unclear, but several theories have been suggested to explain the causes of this phenomenon [18,19]. On the one hand, spasticity can appear as a result of an imbalance of neurotransmitters involved with the alpha motor neurons after damage to the nervous system and related muscles. This imbalance affects the inhibitory and excitatory signals sent to the muscles, causing them to lock in place.

On the other hand, an alternative theory points to the formation of lesions in the upper motor neurons. Once again, the hypothesis is that the flow of muscle contraction signals can be impaired and produce spasticity. The effects on muscles and joints depend on the type of neurological damage. A further description of the causes of spasticity is out the scope of this paper; however, some published papers on the specific topic may help to understand the pathophysiology and underlying mechanism of spasticity [16,17,20].

On account of the above, the formal definition of spasticity has been redefined over the years [10,21]. Originally, it was associated with “a soft yielding resistance that appeared only towards the end of a passive stretch and increased amplitude stretch reflex” [22]. In 1980, Lance suggested defining spasticity as “a motor disorder characterized by a velocity-dependent increase in tonic stretch reflexes (muscle tone) and increased tendon jerks resulting from disinhibition of the stretch reflex, as one component of an upper motor neuron lesion” [2]. Later, other characteristics of upper motor neuron syndrome were added to this definition [10]. More recently, according to the Spasticity Study Group “SPASM” (Support Programme for Assembly of a database for Spasticity Measurement), a more practical definition could be “a sensorimotor control disorder that emerges as a result of upper motor neuron syndrome and in the form of muscles’ involuntary intermittent or permanent activation” [23].

The continuous updates in spasticity definition and the poorly understood of the underlying mechanism highlight the fact that the impact of spasticity is extremely variable, making its management difficult. Hence, the following section presents a brief overview of principal aspects that involve spasticity management. This includes the most commonly used scales to quantify the degree of spasticity and the existing treatments to reduce its aftermaths.

### 2.1. Assessment of Spasticity Degree

Clinical examination of spasticity can be performed in four stages [24]. First, one involves the initial appraisal the clinician performs when the patient enters the examination room, noting the relevant spasticity traits in posture and motion. Stage two involves a detailed examination of the range of joint motion, reflexes, patient’s active motion, among others. Stage three implies an examination of body motor skills. Finally, stage four evaluates the body balance and gait problems for short and long-distance. Note that in all the stages there is a strong one-on-one interaction between the examiner and the patient.

Moreover, complexity when evaluating spasticity lay on the co-occurrence of neural (a velocity-dependent increase in the tonic stretch reflex) and non-neural (e.g., loss of sarcomeres, sub-clinical contractures) causes of increased passive movement resistance [25]. Thus, instrumented and non-instrumented clinical tools are available for measuring the degree of spasticity (see Table 1) [25,26]. The non-instrumented category can be divided into observational and self-reported measures [26], according to the method of reporting the disorder effects by the therapist or patient, respectively.

In the observational category, spasticity can be measured through various scales [27], being the most extended the Ashworth scale and its modified version. The original Ashworth scale contained five grades of measurement and focused on the changes in the resistance to passive movement. The evidence would suggest that the resistance to passive movement is not an exclusive measure of spasticity and is not significantly influenced by reflex-mediated neural activity unless the velocity of passive stretching is high. Ashworth scale is better for the distal muscle groups and poor for the proximal muscle group. Moreover, therapists with greater experience graded spasticity as 1 and 2 more often than the less experienced therapists who used grades 0 and 3 instead. Anyway, the validity of the scale has been tested and its functionality for the upper limb proven [28]. The Ashworth scale was upgraded by Bohannon and Smith, adding a new grade of measure (1+) and rename it as the Modified Asworth scale (MAS). The MAS increased the sensitivity, but incremented the probability of error. Nevertheless, this improvement increased the resolution of the scale, allowing clinicians a better and more precise analysis [29]. Nowadays, the MAS is the most used scale and supports the majority of the studies attempting a more precise evaluation.

Another method of evaluation is using the self-reported scales, where the clinician asks a series of questions about the severity of their symptom that are answered verbally by the patient. Namely, these scales have been developed to capture the patient’s perspective of spasticity. However, these scales are limited by subjectivity, particularly when evaluating interventions where blinding is difficult or not possible [26].

The above approaches, however, are based on perception and provide a subjective outcome, complicated to measure and define [21]. One major drawback of this approach is that the clinician experience is critical in the assessment and during the rehabilitation process. No support is given far from the scales and such manual procedure highlighted a limitation. This evidence presented reveals the need for an objective assessment in patients with spasticity [25].

In this way, a number of instrumented measures of spasticity have been developed in order to increase the precision, validity, reproducibility and objectivity. For that purpose, different sensors are included in the classical assessment procedure. As an example, the instrumented version of the Pendulum scale uses some markers to track the limb motion and measure non-observational parameters like angular displacement, velocity or acceleration. In the case of the instrumented Tardiu scale, the electrophysiological signals and forces during the limb mobilization are captured by Electromyography (EMG) and force/torque sensors, respectively.

On account of the above, the use of instrumented versions of the traditional clinical scales seems promising in order to reduce the limitations of manually-performed procedures [30].

### 2.2. Treatments to Reduce the Spasticity Effects

There are several treatment options for reducing the spasticity impact, and patients usually undergo more than one treatment at a time. As Table 2 depicts, treatments can be sorted in interventional and non-interventional procedures, depending on surgery or not is needed.

Non-surgical treatments that have been shown effective in alleviating symptoms of spasticity include physical therapy, occupational therapy, casting or bracing, oral medications and botulinum toxin (botox) injections. On the contrary, surgery treatments are focused on compensating for imbalance electric stimulus or supplying medication at spinal fluid, denoted as selective dorsal rhizotomy (SDR) and intrathecal baclofen (ITB) therapy, respectively. In the case of the SDR, the goal is to balance the electrical signals (spasticity can be caused by an imbalance in electrical signals to antagonist’s muscles) sent to the spinal cord by cutting selective nerve roots. In the case of the ITB pump, the aim is to decrease spasticity by pump implantation that delivers baclofen to the spinal fluid, directly. This permanent implant causes less severe side effects than oral medicine. Another procedure is the removal of a peripheral nerve to interrupt nerve signal transduction, denoted as neurectomies.

Furthermore, another remarkable non-interventional strategy used to ameliorate the effects of spasticity is robot-aided physical therapy. Currently, there exist numerous robotic systems focused on sensory-motor rehabilitation of patients with motor problems from a neurological disorder; for instance, MIME [31], InMotion [32], NeReBot [33], among others [34,35,36]. Since the use of robotic devices specifically designed for neurorehabilitation has shown positive effects in motor function recovery [37], some studies have included a robotic device for treating the spasticity phenomenon [38]. However, the development of robot-aided systems aiming to evaluate the degree of upper limb spasticity is reduced.

## 3. Literature Review Summary

On account of the above, this paper focused on searching and reviewing studies that pursuit the assessment of UL spasticity with robot-based approaches. The following section presents the results of this systematic review focused on analyzing the characteristics of robot-aided systems for spasticity management. This article does not intend to be a comprehensive analysis of the utility of the robot-aided assessment systems; rather, it aims to compile the information published in peer-reviewed articles.

### 3.1. Search Methods

The authors undertook a literature search until August 2020 about the use of robot-aided systems for the assessment of upper limb spasticity. The keywords used in the search include upper limb, robot, neurological, rehabilitation, assessment, spasticity, motor function, and various combinations of them. The databases consulted were Science Direct, PubMed/Medline, PEDro, Google Scholar and IEEE. Only those articles written in English were considered and duplicated papers were discarded. The criteria for selecting the studies were: (1) systems for the assessment of upper limbs were addressed; (2) systems aided by a robotic device such as exoskeletons or end-effector devices (3) the measure of spasticity was undertaken, and (4) clinical trials with real patients were conducted.

Limitations:the generalisability of these results is subject to certain limitations. For instance, the scope of the study is only the upper limb, but spasticity is presented also in lower limbs. The most affected joint is the ankle [39,40] as it is the elbow on the arm. The scales used for assessment are the same in both limbs, but the effectiveness is different. This systematic review does not lead to meta-analysis. We have inclusion criteria but they have been chosen under the author’s perspective. The table composition will change considerably and the investigation will not guide to the same results varying the criteria. Furthermore, the punctuation estimated in the characteristics of the robotic device row is not rigorous. The authors grant scores based on the investigation impression, but either scope and the points are subjective. However, we will accept these findings, taking into consideration the overall limitations.

### 3.2. Robot-Based Assessment of Spasticity

The results of the systematic review of robot-aided systems for the assessment of UL spasticity are summarized in Table 3. The chosen articles were assigned with different identification numbers (ID) for better explanation throughout the text. The studies were classified according to the addressing or not of relevant aspects of UL assessment such as characterization of arm behavior, reference scales to contrast results, morphology of robotic devices, human–robot interaction, and whether or not dichotomy assessment-training is considered. The above-identified aspects are described in detail as follows.

#### 3.2.1. User’S Arm Behavior Modeling

**Figure 1:**
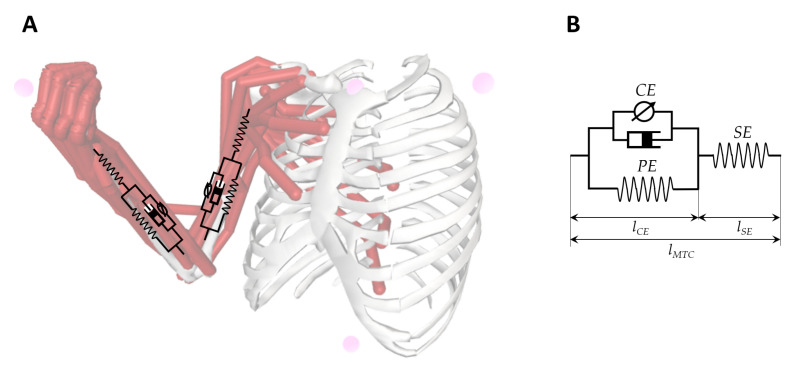
(**A**): Simple biomechanical (dynamic) model of the upper extremity in OpenSim software. (**B**): Schematic drawing of the musculoskeletal model of the arm and Hill-type muscle unit, where contractile element (CE) is a contractile element, parallel (PE) is a parallel elastic element, series (SE) is a series elastic element, $l_{CE}$ is CE length, $l_{SE}$ is SE length.

Proper modeling of the arm behavior is crucial to detect and measure the gap between healthy and spastic muscles. There are a number of models available for representing arm behavior [41]. One of the most well-known models is Hill’s muscle model, which is illustrated in Figure 1. The three-element Hill muscle model is a representation of the muscle mechanical response. The model is constituted by a contractile element (CE) and two non-linear spring elements, one in series (SE) and another in parallel (PE). There are other models, such as the one of Greene and McMahon, or the one of Levin–Wyman modified by Verkhoshansky for spasticity, Hill’s muscle model is the most common representation illustrating not only muscles but it also joints. Accordingly, muscle modeling entails a considerable amount of work and not all studies undertake it.

Despite existing a lot of efforts in the characterization of arm behavior, it can be noted in this review that not all the authors have considered this issue in their studies. Only 20% of studies (IDs: 2,4,6,10,13, and 14) include a model of the arm to related the joint torques and stiffness to the endpoint robot forces and torques.

In any case and despite the great effort of developing new models, the muscular configuration helps us understand the upper limb behavior improving the assessment and subsequent rehabilitation. Furthermore, estimating approaches based on a developed muscle models obtain better accuracy in their final outcome.

#### 3.2.2. Control Strategy

The control strategy performed by the robot can be categorized into: active, passive, assistive, passive-mirrored, active-assistive, corrective, path guidance and resistive [42]. For our literature review, We will focus on the three used in the studies: active, passive and assistive. In active control, the robot is used as a measurement device, without providing force to the subject’s limb. This control strategy is the most used (ID’s: 2, 3, 4, 5, 6, 7, 9, 11, 12, 13, 14, 15, 16, 18, 19, 20, 21, 22, 25, 26, 27, 28, 29) measuring only during the stretching or exercise. In passive control, the robot performs the movement without any account of the subject’s activity. Frequently, this type of control is used to measure the resistance to passive movement and we can find it in (ID’s: 1, 2, 8, 10, 13, 17, 22, 23, 24, 27, 28, 29). In assistive control, the subject’s voluntary activity is required during the entire movement. Robots can assist either providing weight support or providing forces aiming at task completion (ID’s: 1, 2, 9, 10, 11, 14, 15, 21, 22, 26). Generally, the application of this control is combined with others, the active-assistive being the most used.

**Table 3:** Summary of automatic systems for upper limb spasticity assessment.

ID	Source	User’s Arm Behavior Modeling *	User’s Arm Motion & Response Capturing	Control Strategy	Morphology of Robotic Device ${}^{★★}$	Characteristics of Robotic Device ${}^{★}$		User Interfaces (Patient; Therapist)	Approach of Evaluation	Outcome Provided	Correlation Study with	Sample Size	Rehab Mode	Target Human Joint
(1)	Norton, B. (1972) [43]	✘	3 EMG electrodes	Passive-assisted	Exoskeleton (1 DOF)	Accuracy: Portability: Adaptability:	✪✪ ✪✪ ✪✪	P: None T: None	Hysteresis loops (40 s)	Position, force and EMG recordings	None	40 subjects + 3 patients	✘	Elbow (Hemiplegic)
(2)	Reinkensmeyer, D. (1999) [44]	✔	None	Active-assistive + passive	Exoskeleton (ARM Guide) (2 DOF)	Accuracy: Portability: Adaptability:	✪✪ ✪✪ ✪✪	P: None T: A computer	FUGL motor performance exam (Post-processed)	Force patterns	None	4 patients	✔	Shoulder + Elbow (Hemiplegic)
(3)	Pandyan, A. (2001) [45]	✘	None	Active	Exoskeleton	Accuracy: Portability: Adaptability:	✪✪ ✪✪✪ ✪✪✪	P: None T: None	Correlation between observed and measured MAS and RTPM (7 min)	RTPM	MAS	16 subjects	✘	Elbow (Poststroke)
(4)	Lee, H.M. (2004) [46]	✔	1 differential pressure sensor 1 angular rate sensor 2 sensing air bags 1 gyroscope	Active	End-effector	Accuracy: Portability: Adaptability:	✪✪✪ ✪✪✪ ✪	P: None T: None	Real-time	(Velocity-profile graphics)	MAS, UPDRS	15 subjects + 15 patients	✘	Elbow (Poststroke)
(5)	Wu, Y.N. (2004) [47]	✘	2 EMG electrodes	Active	Exoskeleton	Accuracy: Portability: Adaptability:	✪✪ ✪✪✪ ✪✪	P: None T: None	Estimation of velocity-dependent viscous component	Biomechanical and neurophysiological data	MAS	13 patients	✘	Elbow (Poststroke)
(6)	Chen, J.J.J (2005) [48]	✔	1 differential pressure sensor 1 angular rate sensor 2 sensing air bags 1 gyroscope 2 EMG electrodes	Active	Endeffector	Accuracy: Portability: Adaptability:	✪✪✪ ✪✪✪ ✪	P: None T: A computer	Estimation of velocity-dependent viscous component	Biomechanic parameters	MAS	10 patients	✘	Elbow (Chronic stroke)
(7)	Kumar Raj.T.S (2006) [49]	✘	None	Active	Exoskeleton	Accuracy: Portability: Adaptability:	✪✪ ✪✪✪ ✪✪	P: None T: None	Linear regression technique (10 min)	RTPM	MAS	111 patients	✘	Elbow (Poststroke)
(8)	Fazekas, Gabor (2006) [50]	✘	None	Passive	Endeffector (REHAROB) (6 DOF + 6 DOF)	Accuracy: Portability: Adaptability:	✪✪✪ ✪✪ ✪✪	P: Outer shell with handle T: Hardware Control Panel with predefine programming	Training sessions (30 min)	MAS and FIM score	MAS	4 subjets + 8 patients	✔	Shoulder + Elbow (Hemiparetic)
(9)	Pandyan, A (2006) [51]	✘	2 EMG electrodes	Active-assistive	Exoskeleton	Accuracy: Portability: Adaptability:	✪✪ ✪✪ ✪✪	P: None T: None	Flexo-extensions (16.7 s)	MAS, RPE, FEMG	MAS, RPE, FEMG	14 patients	✘	Elbow (Poststroke)
(10)	Nef and Riener (2007) [52]	✔	None	Passive-assistive	Exoskeleton (ARMin)(4 DOF)	Accuracy: Portability: Adaptability:	✪✪✪ ✪✪ ✪✪	P: 1 graphic display for patient T: 1 graphic display for therapist	Mobilisation therapy and ball game therapy (60 min)	Recorded trajectories and 3D disturbance simulations	None	11 patients	✔	Shoulder + Elbow (Hemiplegic and chronic stroke)
(11)	Takahashi Craig.D. (2008) [53]	✘	None	Active-assistive	Endeffector (HWARD) (3 DOF)	Accuracy: Portability: Adaptability:	✪✪✪ ✪✪ ✪✪	P: Computer monitor and + 3 soft straps in hand T: Computer monitor with game difficulty adjusting	Nine different computer games (1.5 h)	MAS, FUGL, ROM, Stroke impact, grasp and pinch force	MAS, FUGL, ROM	13 patients	✔	Hand-wrist (Poststroke)
(12)	Calota and Levin (2008) [54]	✘	2 EMG electrodes	Active	Exoskeleton (Montreal Spasticity Measure)	Accuracy: Portability: Adaptability:	✪✪ ✪✪ ✪✪	P: Not specified T: A Computer	Flexo extensions (5 min)	TSRT	MAS, Tardieu	20 patients	✘	Elbow (Poststroke)
(13)	Bovolenta, F (2009) [55]	✔	None	Active and passive	Endeffector (ReoGo)	Accuracy: Portability: Adaptability:	✪✪✪ ✪✪ ✪✪	P: Computer monitor T: Computer monitor	Nine different computer games (1.5 h)	MAS, FUGL, ROM, Stroke impact, grasp and pinch force	MAS, FUGL, Tardieu	13 patients	✘	Shoulder + Elbow (Poststroke)
(14)	Posteraro, F (2009) [56]	✔	None	Active-assistive	Endeffector (MIT-MANUS) (2 DOF)	Accuracy: Portability: Adaptability:	✪✪✪ ✪✪ ✪✪	P: A display T: Not specified	Robot-assisted therapy (60 min)	CM, MSS, MAS, FUGL, ROM	CM, MSS, MAS, FUGL, ROM	14 patients	✔	Shoulder + Elbow (Hemiparetic)
(15)	Posteraro, F (2010) [57]	✘	None	Active-assistive	Endeffector (MIT-MANUS) (2 DOF)	Accuracy: Portability: Adaptability:	✪✪ ✪✪ ✪✪	P: A display T: Not specified	Robot mediated therapies (45 min)	Motor status core, MAS, ROM	MAS, ROM	34 patients	✔	Shoulder + Elbow (Chronic-hemiparetic)
(16)	Ferreira, J (2011) [58]	✘	1 goniometer Unknown EMG electrodes	Active	Exoskeleton	Accuracy: Portability: Adaptability:	✪✪✪ ✪✪ ✪✪	P: None T: A computer	Linear regression technique (Detection algorithm)	TSRT	None	25 patients	✘	Elbow (Post-stroke + cerebral palsy)
(17)	Fazekas, Gabor (2011) [59]	✘	None	Passive	Endeffector (REHAROB) (6 DOF + 6 DOF)	Accuracy: Portability: Adaptability:	✪✪✪ ✪✪ ✪✪	P: Outer shell with handle T: Hardware Control Panel with predefine programming	Training sessions (30 min)	RMA, MAS, ROM, FUGL and FIM score	RMA, MAS, ROM, FUGL and FIM score	30 patients	✔	Shoulder + Elbow (Hemiparetic)
(18)	Kim, E.H (2011) [60]	✘	None	Active	Endeffector (Hand-stretching device)	Accuracy: Portability: Adaptability:	✪✪ ✪✪✪ ✪✪	P: None T: Not specified	Finger Stretching (10 min)	Mean MAS	MAS	15 patients	✘	Hand (Hemiparetic)
(19)	Hu, X (2013) [61]	✘	4 EMG electrodes	Active	Exoskeleton (2 DOF)	Accuracy: Portability: Adaptability:	✪✪✪ ✪✪✪ ✪✪	P: A table and a sponge T: Only technician Not developed yet	Training sessions (EMG-triggered algorithm) (30 min)	EMG samples and FUGL, MAS, ARAT and WMFT	FUGL, MAS, ARAT and WMFT	10 patients	✔	Hand-wrist (Chronic-stroke)
(20)	Ferreira, J (2013) [62]	✘	1 electrogoniometer Unknown EMG electrodes	Active	Exoskeleton	Accuracy: Portability: Adaptability:	✪✪✪ ✪✪ ✪✪	P: None T: A computer	Passive muscle stretch at different velocities (Detection algorithm)	TSRT	None	11 patients	✘	Elbow (Post-stroke + cerebral palsy)
(21)	Sale and Posteraro (2014) [63]	✘	None	Active-assistive	Endeffector (MIT-MANUS) (2 DOF)	Accuracy: Portability: Adaptability:	✪✪ ✪✪ ✪✪	P: A display T: Not specified	Robot-assisted therapy (45 min)	MAS-S, MAS-E, pROM	MAS-S, MAS-E, pROM	53 patients	✔	Shoulder + Elbow (Subacute stroke)
(22)	Taveggia, G (2016) [64]	✘	None	Active-assitive and passive	Exoskeleton (ARMEO spring)	Accuracy: Portability: Adaptability:	✪✪✪ ✪✪ ✪✪	P: A display T: A computer	Training sessions (SPSS software) (60 min)	Motricity index, MAS and NPRS	MAS	54 patients	✘	Shoulder + Elbow + Wrist (Poststroke)
(23)	Pennati, G.V (2016) [65]	✘	None	Passive	Exoskeleton (NeuroFlexor) (1 DOF)	Accuracy: Portability: Adaptability:	✪✪ ✪✪✪ ✪✪	P: A display T: A digital	Estimation of Neural and Viscous component	Cut-off values	MAS, FUGL	107 patients	✘	Wrist (Poststroke)
(24)	Dehem, S (2017) [66]	✘	None	Passive	Endeffector (REAplan)	Accuracy: Portability: Adaptability:	✪✪ ✪✪✪ ✪✪	P: A display T: Not specified	Correlation between velocity and RF	Velocity-force graphics	MAS	12 patients	✘	Elbow (Chronic stroke)
(25)	Lee, D.J. (2017) [67]	✘	1 dynamometer	Active	Exoskeleton (1 DOF)	Accuracy: Portability: Adaptability:	✪✪ ✪✪ ✪✪	P: Not developed yet T: Computer monitor and a emergency switches to stop	Stretching sessions (90 s)	Force patterns	MAS	9 patients	✔	Elbow + Hand-wrist (Poststroke)
(26)	Calabro, R.S (2017) [68]	✘	3 EMG electrodes	Active-assistive	Exoskeleton (Armeo power) (6 DOF)	Accuracy: Portability: Adaptability:	✪✪✪ ✪✪✪ ✪✪	P: A display T: Not specified	Training sessions (EMG Algorithm Shapiro-Wilk statistic) (60 min)	MAS, FUGL	MAS, FUGL	20 patients	✔	Shoulder + Elbow (Ischemic stroke)
(27)	Posteraro, F (2018) [69]	✘	None	Active and passive	Exoskeleton (NEUROExos Elbow Module) (4 DOF)	Accuracy: Portability: Adaptability:	✪✪✪ ✪✪ ✪✪	P: Not specified T: Not specified	Isokinetic passive mobilization (45 min)	MAS score	MAS	5 patients	✔	Elbow (Poststroke)
(28)	Wang, H. (2019) [12]	✘	None	Active and Passive	End-effector (Humac Norm) (1 DOF)	Accuracy: Portability: Adaptability:	✪✪ ✪✪ ✪✪	P: A display T: PC interface	Online	Peak torque; Keep time; Rise time	MAS	14 patients (stroke)	✘	Elbow
(29)	Sin, M. (2019) [70]	✘	1 EMG	Active and Passive	End-effector (1 DOF)	Accuracy: Portability: Adaptability:	✪✪ ✪✪✪ ✪✪	P: Not specified T: Not specified	Manual and Isokinetic mobilization (37 min)	Intraclass correlation coefficient	MAS, MTS	17 patients (stroke)	✘	Elbow

* Considering the automatic administration of the test(✔ = Yes; ✘ = No); ^★^ Given in levels (Low: ✪, Medium: ✪✪, High:✪✪✪); ^★★^ Exoskeleton or Endeffector.

#### 3.2.3. Morphology of Robotic Devices

**Figure 2:**
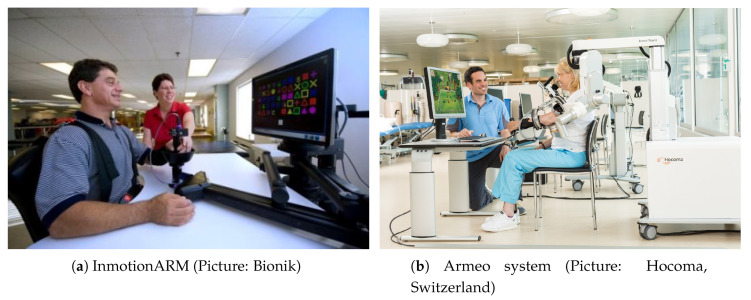
Systems for upper limb rehabilitation, commercially available.

Robot-aided limb mobilization is an extended strategy towards reducing the inter-operator variability in spasticity assessment. For that purpose, two main types of devices are employed: end-effector or exoskeleton robots. We denoted as end-effector devices those in which the contact point between human and robot is the robot’s tip (hand-held). This is an easily releasable setup that increases the safeguarding levels. Moreover, this group of collaborative robots does not require a safety space and therapist, patient and robot can share the same space (IDs: 4, 6, 8, 11, 13, 14, 15, 17, 18, 21, 24, 28, 29). Figure 2a illustrates an example of an end-effector system, the InMotion Arm.

Exoskeletons are wearable devices that attach and adjust to the patient’s upper limb, passively mobilizing the arm. We identify the use of exoskeletons in two main configurations: fixed or added. On the one hand, the first configuration includes the robots which have a solid base, a structure from the ground and finally the adjustable arm tool. Usually, they are fixed in a specific room but sometimes the base has wheels to ease the movement. The exoskeleton provides a more precise measure but the robot management is more difficult. On the other hand, the added robots only consist of an adjustable arm tool. Easy interchange between patients gives a quick assessment and achieves a complete rehabilitation (IDs: 1, 2, 5, 7, 9, 10, 12, 16, 19, 20, 23, 25, 26, 27). Still, a major disadvantage is the robot fixation on the patient’s upper limb. A device malfunction could provoke physical damage into the already injured arm with no possibility of release. Figure 2b illustrates an example of the exoskeleton system, the Armeo Power.

The characteristics of the robotic device depends on its morphology. The principal advantage of the exoskeleton is control. Because of the rigid structure, exoskeleton systems do not need an additional measuring sensor. The range of motion is higher in comparison with the endeffector systems. For this reason and generally, exoskeleton devices will have better accuracy and portability than endeffector systems.

#### 3.2.4. User Interfaces

In a robot-aided therapy, three participants of the process can be appreciated: the therapist, patient, and robotic device. Therefore, the interplay between these actors can be analyzed from two perspectives: between therapist and robot (T-R) and between patient and robot (P-R).

Initially, robot-assisted devices focused their efforts on developing a precise assessment or effective rehabilitation (IDs: 1, 3, 4, 5, 7), while leaving by the wayside to include proper user interfaces. Basically, the device was fully controlled by a technician specialized in this robot and he explained the basics to the therapist. In isolated cases, the user had a computer screen but it was not an extended practice.

Fortunately, this approach evolved with the technology and we can see a tendency where the overall experience is a key factor. The interaction, not only with the patient, but also with the therapist, remodeled the predefined designs adding new elements to develop. The usual and most common tool to connect humans with robots is a display. A screen, touchable or not touchable, support visual learning by imitation and creates a bond between human and machine. Furthermore, any kind of graphic information could be shown on the screen such as rehabilitation videos, serious games, real-time measures, or just entertainment. The patient pursues better a rehabilitation process based on games or entertainment than usual flexo-extensions (IDs: 10, 11, 13).

Furthermore, the therapist can also personalize better the rehabilitation routine with visual support. Usually, clinicians are not technical experts, and their knowledge of the device is limited. Understanding the machine options and restrictions is fundamental for effectivley exploiting the possibilities of the tool. Then, like the patient, the therapist is an important target to consider in the value chain (IDs: 8,10,11,13,17,25,28).

#### 3.2.5. Approach of Evaluation

Many researchers have developed a number of techniques to evaluate spasticity. We could encompass all methods into three main categories: robot-assisted mobilization, predictive approaches and more analytical procedures based on cutting-edge algorithms. In the first category, we identify the systems with an evaluation using therapy and rehabilitation (ID’s: 8, 9, 10, 11, 12, 13, 14, 15, 17, 19, 21, 22, 25, 26, 27, 28, 29). This method proposes a primary evaluation, then a therapy process with exoskeleton or endeffector and a final reevaluation. The main advantage of this approach is the use of known scales as the MAS or FMA to achieve the primary evaluation. At the end of the therapy, an improvement tendency can be noticed in most patients. However, the original outcome is subjective and the final assessment too. In the second category, system evaluation is done through estimation of the viscous components (ID’s: 5,6,23). The estimation of the viscous component is based on the relation between the externally imposed joint displacements and the corresponding joint resistance, which is generally modeled as a second-order system. The effectiveness of this approach depends on the acquisition method and the final outcome deviance from each study. The third category encloses the systems based on algorithms (ID’s: 1,3,7,16,20,24). A detection algorithm monitors the results and return an outcome. The most common technique used is linear regression. Linear regression attempts to model the relationship between two variables by fitting a linear equation to observed data. In particular, velocity and time are measured in each mobilization and with this linear trend, Resistance to Passive Movement (RPM) is quantified. Another type of algorithm used is the hysteresis loops. During a movement cycle, an XY recorder plots force versus position and display the data in the form of a hysteresis loop. A complete absence of muscular resistance produces identical traces in both directions.

The role of the robots in all the approaches is capturing data. Afterwards, the objective data collected is processed and supports the final clinician evaluation. All three techniques are effective, but predictive approaches and more analytical procedures based on cutting-edge algorithms provide an estimation from the capture data while robot-assisted rehabilitation-only measure the variation between the original and the final status.

#### 3.2.6. Outcome Provided

In order to assess spasticity, different outcomes can be obtained from robot-aided interventions. We encountered from velocity-force graphics (ID: 24) to recorded trajectories (ID: 10). The outcome provided by the robot is numerical or graphical, but is usually in mathematical form. These data must be interpreted with caution because minimal variation can lead to a considerable decreasing or increasing muscle tone.

There are currently two major approaches being adopted in spasticity evaluation. One is through rehabilitation and the existing scales and the other one is estimating the tonic stretch reflex threshold (TSRT). In the first approach, the system of assessment consists of a early evaluation with known scales such as MAS, Fugl–Meyer assessment, Functional independent Measure, Unified Parkinson’s disease rating scale or Rivermead motor assessment scale (IDs: 8, 9, 11, 13, 14, 17, 19, 21, 26, 27). After passive extremity mobilization or game therapy with training sessions, the scales are matched again.

In the second approach, the velocity-dependent increase is measured. Usually, The TSRT value is estimated using a regressing analysis, interpolating a regression line between each dynamic stretch reflex threshold (DSRT) (IDs: 12, 16, 20, 24). Furthermore, a third minor approach supports the estimation of the velocity-dependent viscous component (IDs: 5, 6, 23). This system of evaluation is more scientific since it is based on arm modeling. During the stretch and utilizing known reactive torque value, angular frequency and derived phase lag, the viscous components are estimated.

Therefore, which is the correlation between, for example, a force pattern to the MAS clinical values? How do we prove its validity? In this regard, the most extended tendency is to build a comprehensive model of reference. As an example, some studies examined healthy patients and determined cut-off values (ID:24) and other investigations estimated it indirectly (ID:26). This second idea used multiple therapists and build the device on their experience. As we explained previously, other studies estimated a known scale value and then matched with a first measure to prove their effectiveness. In this sense, it can be appreciated a high predominance (72%) of small samples of patients in feasibility studies. Only 14% of studies have recruited between 30 and 50 participants, and also the 14% of reviewed studies considered samples of more than 50 participants. This fact may be explained by the heterogeneity in the characteristics of patients with a neurological disorder, which makes it difficult for the recruitment process.

#### 3.2.7. Dual-Operation: Rehab- and Eval-Modes

Finally, we can identify a new approach for developing robotic systems with a dual goal, which is to support not only the assessment stage but also rehabilitation. A high number of studies (41%) have considered this dichotomy, existing an equal participation of exoskeleton (IDs: 2, 10, 19, 25, 26, 27) and end-effector (IDs: 8, 11, 14, 15, 17, 21) devices. Under this paradigm, a robotic device can be used for measuring the degree of spasticity at the beginning of treatment, and subsequently, the same robotic device can execute the treatment protocol in the rehabilitation phase. In this case, the therapist uses only one device for both tasks, having some advantages in clinical practice (e.g., ease of use, cost reduction, optimization of the workspace). Additionally, as mentioned before, a combined rehabilitation process between training exercises with pharmacological approaches (Baclofen or BonT-A injections) shortens the expected rehabilitation time.

#### 3.2.8. Target Human Joints

The human joint classification helps distinguish the different joint targets in each research. The elbow is the most studied joint (ID’s: 1, 2, 3, 4, 5, 6, 7, 8, 9, 10, 12, 13, 14, 15, 16,17, 20, 21, 22, 23, 24, 25, 26, 28, 29), followed by the shoulder (ID’s: 2, 8, 10, 13, 14, 15, 17, 21, 22, 26) and finally the hand-wrist (ID’s: 12, 19, 20, 23, 24, 26). This approach may be explained by the fact that the hand is the most complicated system in the arm. On the contrary, the elbow is easy to move and a developed industrial system could be used to mobilize it. There is no need for a specific tool and passive mobilization is less dangerous in the elbow than in the hand or the wrist.

## 4. Current Status of Robot-Aided Spasticity Assessment Systems

The results of this literature review are quite revealing in several ways. First, we might have identified a growing tendency in the use of techniques for data processing. Initially, researchers did not include any data treatment, but from 2009, nearly all the new investigations are incorporating statistical software or predictive algorithms [71]. This result may be explained by the fact that we are accumulating a large amount of data, and during the last ten years, algorithms were created to manage this considerable volume and to find patterns in the big data [72]. Another important finding was that most of the developed devices matched their outcomes with the MAS scale. A possible explanation for this might be that the MAS is the most used scale among clinicians. The scale behaves better for upper limbs than for lower limbs, but even so, it is widely applied for measuring spasticity in the lower limb [38].

One interesting finding is that similar to data processing, therapist and patient interfaces have been evolving. Originally, no element between the patient and the device was present. Only a small computer with minor possibilities of customization was included for the therapist in some cases. Luckily, this relationship has been improved, new adapting tools for each patient have been added and new technologies as virtual reality (VR) have demonstrated their validity [73]. The device is not alone and additional inputs have been captured from external sensors [74]. Despite these promising advantages, further work is required to enhance the connection between machine, therapist, and patient. Closer inspection of the table could show a tendency in the rehabilitation utility. The devices do not only measure but also rehabilitate. This evidence is important because the patient will interact with only one device and the therapist will only handle one machine [75].

Nevertheless, the steady decline of arm modeling can thus be suggested. None of the last 14 studies focused their efforts on improving the actual arm model. This result is somewhat counterintuitive because a better understanding of arm behavior will guide to better research [76]. Additionally, the size of the patient samples in the studies is often small. This finding could be explained because of the strong regulation in the personal privacy data and more specifically in the clinical data.

In general, it seems that a high number of robots have been designed in the last 15 years for supporting the clinician diagnosis, and the tendency seems to continue the following years with the Healthcare 4.0 concept, which extends the basis of Industry 4.0 in a scenario where patients and healthcare professionals are strictly correlated with the organization, the methodology and the technology [77]. Besides, real-time data will upload from the robot to the patient electronic file, and connectivity will enlarge the user experience, allowing the patients to follow their clinical status at any time from multiple devices. Thus, the therapist could conduct the complete process from a different place than the patient and the robot, adjusting the following sessions with the data acquired. A more precise evaluation will guide to better therapist planning. In order to reach this ideal panorama of smart rehabilitation, some concerns must be addressed.

## 5. Prospects for Improvement in Robot-Aided Upper Limb Spasticity Assessment

Rehabilitation technology and automation of manual processes have become an essential part of rehabilitation development. In this way, the traditional rehabilitation cycle is being transformed into a more autonomous process, denoted as the “automated rehabilitation cycle” [37]. Here, the clinicians are supported by several automated systems in daily activities. This automated rehab cycle is composed of automated assessment systems (AAS), decision support systems (DSS), and robotic rehabilitation systems (RRS). In this new holistic paradigm of rehabilitation, the AAS plays an essential role since they are used at the beginning of treatments to measure the level of functional impairment, and at the end of treatment to determine the effectiveness of therapy. In this context, the robot-aided systems for the assessment of upper limb spasticity fit into the AAS category.

**Figure 3:**
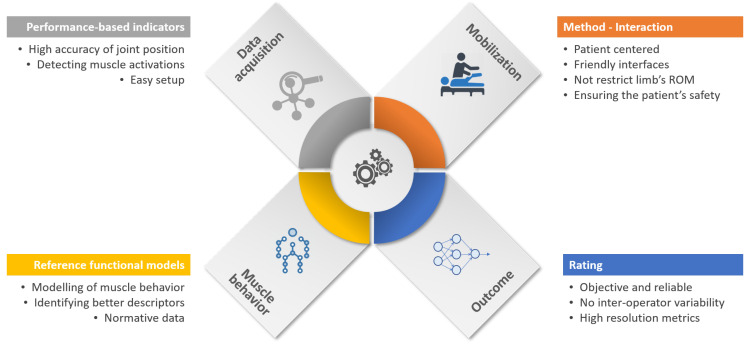
Essential components for robot-aided assessment of upper limb (UL) spasticity.

Intending to obtain an objective evaluation, Figure 3 illustrates the principal components that intervene in the robot-aided assessment process. The first component consists of capturing the relevant data from sensors from the robot or patients. The second component is related to the limb mobilization using the robotic device. Another relevant component is the modeling of arm behavior in order to quantify the degree of impairment. This reference model is created from the robot input, taking into consideration cut-off values from a healthy arm. The last component is related to provide the spasticity descriptor, which ideally might be reliable, robust, and with high resolution.

Although it is clear that stages which participate in the process of evaluation, from data acquisition to outcome generation, new developments must consider some challenges and technical requirements in order to increase the level of automation and better use of gathered data. On account of the above, we encompass what we think future improvements are into three big fields: security, results’ analysis, and a standard evaluation methodology.

### 5.1. Safety in Human-Robot Interactions

In clinical evaluations, security is one of the most important aspects for focus. A robotic device has to increase the security levels to the maximum because a minor malfunction could lead to non-reversal damage. The new collaborative robots remove the physical barriers and share the space between therapist, patient and device with no additional countermeasure. Hence, the use of collaborative robots in clinical settings could increase the safeguarding during physical human-robot interaction. The new soft robotics devices [78] could reduce an occasional and unintentional accident. This technology has been slightly explored in rehabilitation, even considering some types of exoskeletons (cable-driven) as soft or compliance-based devices. Thus, more research is needed in exploiting the application of soft robotics for rehabilitation and assessment of motor function.

On this basis, considering the great efforts of research in robots for healing applications, there is a clear need for regulations [79]. Currently, there exist various standardization institutions dealing with the safety of human interaction with rehabilitation robots. The most influential ones are the International Organisation for Standardisation (ISO) and the American National Standards Institute (ANSI). The goal of this work is to generate dedicated standards for rehabilitation robotics devices. As an example, a relevant standard is the IEC 80601-2-78:2019. Giving a glance into the standard, it can be noted that the most relevant functional requirements for safe robotic systems are related to limiting the forces, speed, and power of the robot. That is, strategies not related to hardware, but software. Consequently, we required the development of more intelligent strategies of control in order to cover the demanded safety levels [80]. For that purpose, artificial intelligence (AI) could be a key component in order to develop self-adaptive algorithms of control.

### 5.2. Intelligent Data Analysis Capabilities

A natural progression of this work is to analyze the new algorithms used in big data as machine learning, artificial intelligence and neuronal networks. The standard model will adjust better to the patient’s necessities and all the patient’s data would build a database, supporting a predictive rehabilitation and an earlier syndrome detection. Furthermore, a precise Tonic stretch reflex threshold will not only be convenient for spasticity assessment but also for other neurodegenerative disorders as Parkinson’s, Huntington’s or Alzheimer’s. Moreover, new equipment as EMG’s, Kinect camera or infrared sensor supports the measurement of the robot adding accuracy to the evaluation. Still, the sensorization demands a postprocessing and interpretation, matching the isolated data with the robot’s.

In this sense, one source of weakness identified in this study is, for instance, the MAS scale dependency [21]. Since 1987, the scale has been present in any new development device. The technology has evolved exponentially the last 30 years and the challenge now is to create a machine able to objectively measure spasticity. Without diminishing the contribution of the MAS scale, a percentage outcome will better support the clinician’s perception and it will build a numeric solid base for future improvements.

Various studies have pointed to the limitations of using five-point ordinal scales to detect minor improvements in motor recovery [21,81]. In this regard, the development of robot-aided assessment systems must consider including methods to increase the resolution of scores towards obtaining high-resolution descriptors of spasticity.

### 5.3. Standardization of Robot-Aided Procedures

Different types of methods have been developed to perform a robot-assisted rehabilitation and every new device introduce an additional method [82,83]. There are a number of similarities between them and part of the aim of this literature review is to identify the relevant common requirements in robot-assisted assessment towards proposing a standard model of evaluation. Thus, in the authors’ opinion, three principal research lines that require improvement in order to generalize the spasticity assessment process exist: the procedure (stages), intensity, and provided metrics.

The first step in this process is the posture. The patient should be sitting on a chair with the upper limbs resting along the body. Thus, the therapist will execute a complete elbow flexo-extension without difficulty, from maximum extension to maximum contraction. If this posture is not physically achievable, the patient will lie on a bed. allowing the therapist the same movement described before. Posture is one of the most important aspects of the method. If the system does not have a compensation procedure, data acquisition could mislead. In our review, only (ID’s: 2, 25) proposes a strap to fix the patient’s trunk to the back of the chair to limit compensatory movements. In other studies, (ID’s: 5, 7) measure/limit the hand movement with two sensing airbags. The ideal system will include equipment (cameras, infrared sensors placed on the arm) to detect compensation movements such as trunk movements, shoulder movements or, on the contrary, the patient should be firmly tied to avoid inaccurate measures.

A vast majority of studies have assessed the efficacy of robot-assisted rehabilitation [84,85,86] and the conventional number of repetitions in the investigated articles is 3.2 min minimum between repetitions is required to favor the muscle recovery and a maximum of 45 min sessions recommended.

Up to now, previous studies have used diverse metrics. As shown in Table 3, there is no common outcome in the 28 studies, but still, some similarities have been found. The MAS scale is present in most researches [87], moreover, TSRT [88] and the resistance to passive movement (RTPM) are also proper indicators to classify the spasticity grade. Taken together, these suggested steps establish a standard procedure for any future robot-assisted rehabilitation device.

## 6. Conclusions

Rehabilitation robotics comprises one of the fields that has grown steadily in recent decades. However, the main focus of research has been the development of systems to assist or improve intervention stages. Thus, a minor development of automatic assessment systems is identified, especially for UL spasticity. In this paper, a total of 28 studies focused on the automatic assessment of UL spasticity using a robotic device was reviewed. From the comprehensive analysis of the above studies, various limitations and challenges in the development of optimal assessment systems for UL spasticity have been identified.

Firstly, a significant issue to continue improving is the development and standardization of effective strategies to guarantee the patient’s safety during robot-aided limb mobilization. In this way, the use of collaborative robots that are considered intrinsically safe systems could be a feasible alternative to end-point devices. In the case of exoskeleton-based strategies, the integration of recent advances in soft robotic technology can enhance the comfort and safeguarding of patients by default.

Secondly, the evaluation of the degree of spasticity throughout the entire range of joint motion is needed. In some cases, the morphology of devices or the administration setup can reduce the complete range of joint motion. Therefore, it is essential that the characteristics of the robotic device would not restrict the whole range of motion of the target joint.

It is necessary that the collaboration between researchers and medical practitioners in order to build appropriate biomechanical models of reference (dynamic and kinematic) to better identify the gap between physical movement and theoretical patterns of motion. More comprehensive reference models of spastic muscle behavior would allow understanding of the extent of the functional limitations derived from spasticity.

In this sense, it is also important to stress better usage of gathered data from robotic and automatic systems in order to increase the resolution of the spastic descriptors. Currently, five-point ordinal scales for scoring the degree of spasticity are still the golden standard. However, the analysis of the richer performance-based data measured by robotic systems can lead to more descriptive spasticity outcomes, and consequently, to optimally tailored protocols of rehabilitation.

For concluding, it is our opinion that the benefits offered by robot-aided assessment systems can complement the holistic rehabilitation cycle and that these kinds of systems will become a complementary tool in daily clinical practice.

## Figures and Tables

**Table 1:** Overview of procedures for measuring spasticity according to topology.

Category	Scale	Principle	Outcome
Observational	Ashworth Scale	Rating the resistance to manually limb mobilization	0–4 scale (1-point extra in modified version)
	Tardieu Scale	Rating the resistance to manually limb mobilization and the angle where this resistance occurs	0–4 scale (1-point extra in modified version) + Two angles (R1, R2)
	Pendulum test	Observing a muscle’s response and oscillations to sudden stretch imposed by gravity	There is no accepted scale (observation-based rating)
	Tone Assessment Scale (TAS)	Evaluating the resting posture, the response to passive movement and the response to active efforts (multi-item)	0–4 scale
Self-reported	Penn Spasm Frequency Scale	Counting the number of spasms experienced in a specified time frame	0–4 scale
	Numeric Rating Scale (NRS)	Self-appreciation of severity of their symptoms	0–10 scale
Instrumented	Ultrasound muscle elastography	Examining the mechanical elastic properties of tissues	5-point scale
	Instrumented Hofmann’s reflex	Measuring the threshold spinal reflex reaction by electromyography (EMG)	H-reflex
	Instrumented Pendulum Scale	Markers are adhered to limb and the trial is videotaped to allow computerized motion analysis	Angular displacement, velocity, and acceleration response
	Instrumented Tardieu Scale	Integrating biomechanical and electrophysiological signals during limb mobilization	Joint angle and torque + surface electromyography

**Table 2:** Overview of treatments for spasticity.

Category	Procedure	Aim
Non-interventional	Physical therapy	Improving movement
	Occupational therapy	Improving autonomy in ADL
	Casting or bracing	Reducing secondary damage
	Pharmacological (oral medication and injections)	Improving movement
Interventional	Selective dorsal rhizotomy (SDR)	Balancing electrical stimulus
	Intrathecal baclofen (ITB) pump	Supplying medication at spinal fluid
	Neurectomies	Removal damaged nerves

## Abbreviations

The following abbreviations are used in this manuscript:

MAS	Modified Ashworth Scale
RTPM	Resistance to passive movement
TSRT	Tonic stretch reflex threshold
FMA	Fugl-Meyer Assessment
MTS	Modified Tardieu Scale
ROM	Range of Motion
FIM	Functional independent Measure
EMG	Electromyography
RMA	Rivermead motor assessment scale
UPDRS	Unified Parkinson’s Disease Rating Scale
FEMG	Free-running electromyography
MSS	Multiple sclerosis-related spasticity
CM	Chedoke–McMaster Stroke Assessment
ARAT	Action Research Arm Test
WMFT	Wolf Motor Function Test
MAS-S	Modified Ashworth Scale-Shoulder
MAS-E	Modified Ashworth Scale-Elbow
pROM	Total Passive Range of Motion-Shoulder/Elbow
NPRS	Numeric pain rating scale
VR	Virtual reality
DOF	Degrees of freedom

## References

[1] Biering-Sørensen F., Nielsen J.B., Klinge K. (2006). Spasticity-assessment: A review. Spinal Cord.

[2] Lance J.W. (1980). The control of muscle tone, reflexes, and movement: Robert Wartenbeg Lecture. Neurology.

[3] Kuo C.L., Hu G.C. (2018). Post-stroke Spasticity: A Review of Epidemiology, Pathophysiology, and Treatments. Int. J. Gerontol..

[4] Rekand T. (2010). Clinical assessment and management of spasticity: A review: Clinical assessment and management of spasticity. Acta Neurol. Scand..

[5] Kwakkel G., Meskers C.G.M. (2015). Botulinum toxin A for upper limb spasticity. Lancet Neurol..

[6] Thibaut A., Chatelle C., Ziegler E., Bruno M.A., Laureys S., Gosseries O. (2013). Spasticity after stroke: Physiology, assessment and treatment. Brain Inj..

[7] Kinnear B.Z., Lannin N.A., Cusick A., Harvey L.A., Rawicki B. (2014). Rehabilitation Therapies After Botulinum Toxin-A Injection to Manage Limb Spasticity: A Systematic Review. Phys. Ther..

[8] Hesse S., Reiter F., Konrad M., Jahnke M.T. (1998). Botulinum toxin type A and short-term electrical stimulation in the treatment of upper limb flexor spasticity after stroke: A randomized, double-blind, placebo-controlled trial. Clin. Rehabil..

[9] Hoare B.J., Imms C. (2004). Upper-Limb Injections of Botulinum Toxin-A in Children With Cerebral Palsy: A Critical Review of the Literature and Clinical Implications for Occupational Therapists. Am. J. Occup. Ther..

[10] Petek Balci B. (2018). Spasticty measurement. Arch. Neuropsychiatry.

[11] Sherwood A., McKay W.B., Akay M. (2006). Spasticity and Upper Motor Neuron Dysfunction. Wiley Encyclopedia of Biomedical Engineering.

[12] Wang H., Huang P., Li X., Samuel O.W., Xiang Y., Li G. (2019). Spasticity Assessment Based on the Maximum Isometrics Voluntary Contraction of Upper Limb Muscles in Post-stroke Hemiplegia. Front. Neurol..

[13] Johnson G.R. (2002). Outcome measures of spasticity. Eur. J. Neurol..

[14] Ashworth N., Satkunam L., Deforge D., The Cochrane Collaboration (2004). Treatment for spasticity in amyotrophic lateral sclerosis/motor neuron disease. The Cochrane Database of Systematic Reviews.

[15] Walton K. (2003). Management of Patients With Spasticity-A Practical Approach. Pract. Neurol..

[16] Li S., Francisco G.E. (2015). New insights into the pathophysiology of post-stroke spasticity. Front. Hum. Neurosci..

[17] Trompetto C., Marinelli L., Mori L., Pelosin E., Currà A., Molfetta L., Abbruzzese G. (2014). Pathophysiology of spasticity: Implications for neurorehabilitation. Biomed Res. Int..

[18] Priori A., Cogiamanian F., Mrakic-Sposta S. (2006). Pathophysiology of spasticity. Neurol. Sci..

[19] Ward A.B. (2012). A literature review of the pathophysiology and onset of post-stroke spasticity. Eur. J. Neurol..

[20] Mukherjee A., Chakravarty A. (2010). Spasticity mechanisms–for the clinician. Front. Neurol..

[21] Malhotra S., Pandyan A., Day C., Jones P., Hermens H. (2009). Spasticity, an impairment that is poorly defined and poorly measured. Clin. Rehabil..

[22] Denny-Brown D. (1966). The Cerebral Control of Movement.

[23] Burridge J., Wood D., Hermens H.J., Voerman G., Johnson G., Wijck F.V., Platz T., Gregoric M., Hitchcock R., Pandyan A. (2005). Theoretical and methodological considerations in the measurement of spasticity. Disabil. Rehabil..

[24] Petropoulou K. (2017). Spasticity - Management with a Focus on Rehabilitation. International Neuromodulation Society. https://www.neuromodulation.com/fact_sheet_spasticity.

[25] Aloraini S.M., Gäverth J., Yeung E., MacKay-Lyons M. (2015). Assessment of spasticity after stroke using clinical measures: A systematic review. Disabil. Rehabil..

[26] Hugos C.L., Cameron M.H. (2019). Assessment and Measurement of Spasticity in MS: State of the Evidence. Curr. Neurol. Neurosci. Rep..

[27] Platz T., Eickhof C., Nuyens G., Vuadens P. (2005). Clinical scales for the assessment of spasticity, associated phenomena, and function: A systematic review of the literature. Disabil. Rehabil..

[28] Lee K.C., Carson L., Kinnin E., Patterson V. (1989). The Ashworth Scale: A Reliable and Reproducible Method of Measuring Spasticity. Neurorehabilit. Neural Repair.

[29] Pandyan A.D., Johnson G.R., Price C.I.M., Curless R.H., Barnes M.P., Rodgers H. (1999). A review of the properties and limitations of the Ashworth and modified Ashworth Scales as measures of spasticity. Clin. Rehabil..

[30] Oña Simbaña E.D., Sánchez-Herrera Baeza P., Jardón Huete A., Balaguer C. (2019). Review of Automated Systems for Upper Limbs Functional Assessment in Neurorehabilitation. IEEE Access.

[31] Lum P.S., Burgar C.G., Loos M.V.d., Shor P.C., Majmundar M., Yap R. (2006). MIME robotic device for upper-limb neurorehabilitation in subacute stroke subjects: A follow-up study. J. Rehabil. Res. Dev..

[32] Volpe B.T., Lynch D., Rykman-Berland A., Ferraro M., Galgano M., Hogan N., Krebs H.I. (2008). Intensive Sensorimotor Arm Training Mediated by Therapist or Robot Improves Hemiparesis in Patients With Chronic Stroke. Neurorehabilit. Neural Repair.

[33] Rosati G., Gallina P., Masiero S. (2007). Design, Implementation and Clinical Tests of a Wire-Based Robot for Neurorehabilitation. IEEE Trans. Neural Syst. Rehabil. Eng..

[34] Kung P.C., Ju M.S., Lin C.C.K. Design of a forearm rehabilitation robot. Proceedings of the 2007 IEEE 10th International Conference on Rehabilitation Robotics.

[35] Fonseca L.A., Grecco L.A.C., Politti F., Frigo C., Pavan E., Corrêa J.C.F., Oliveira C.S. (2013). Use a Portable Device for Measuring Spasticity in Individuals with Cerebral Palsy. J. Phys. Ther. Sci..

[36] Lunenburger L., Colombo G., Riener R., Dietz V. Clinical Assessments Performed During Robotic Rehabilitation by the Gait Training Robot Lokomat. Proceedings of the 9th International Conference on Rehabilitation Robotics, ICORR 2005.

[37] Oña E.D., Cano-de-la Cuerda R., Sánchez-Herrera P., Balaguer C., Jardón A. (2018). A review of robotics in neurorehabilitation: Towards an automated process for upper limb. J. Healthc. Eng..

[38] Zhou Z., Sun Y., Wang N., Gao F., Wei K., Wang Q. (2016). Robot-Assisted Rehabilitation of Ankle Plantar Flexors Spasticity: A 3-Month Study with Proprioceptive Neuromuscular Facilitation. Front. Neurorobot..

[39] Abu-Dakk F.J., Valera A., Escalera J., Vallés M., Mata V., Abderrahim M., Liu H., Kubota N., Zhu X., Dillmann R., Zhou D. (2015). Trajectory Adaptation and Learning for Ankle Rehabilitation Using a 3-PRS Parallel Robot. Intelligent Robotics and Applications.

[40] Bucca G., Bezzolato A., Bruni S., Molteni F. (2009). A Mechatronic Device for the Rehabilitation of Ankle Motor Function. J. Biomech. Eng..

[41] Adewusi S., Rakheja S., Marcotte P. (2012). Biomechanical models of the human hand-arm to simulate distributed biodynamic responses for different postures. Int. J. Ind. Ergon..

[42] Basteris A., Nijenhuis S.M., Stienen A.H., Buurke J.H., Prange G.B., Amirabdollahian F. (2014). Training modalities in robot-mediated upper limb rehabilitation in stroke: A framework for classification based on a systematic review. J. Neuroeng. Rehabil..

[43] Norton B.J., Bomze H.A., Chaplin H. (1972). An Approach to the Objective Measurement of Spasticity. Phys. Ther..

[44] Reinkensmeyer D., Dewald J., Rymer W. (1999). Guidance-based quantification of arm impairment following brain injury: A pilot study. IEEE Trans. Rehabil. Eng..

[45] Pandyan A., Price C., Rodgers H., Barnes M., Johnson G. (2001). Biomechanical examination of a commonly used measure of spasticity. Clin. Biomech..

[46] Lee H.M., Chen J.J.J., Ju M.S., Lin C.C.K., Poon P.P. (2004). Validation of portable muscle tone measurement device for quantifying velocity-dependent properties in elbow spasticity. J. Electromyogr. Kinesiol..

[47] Wu Y.N., Huang S.C., Chen J.J.J., Wang Y.L., Piotrkiewicz M. (2004). Spasticity Evaluation of Hemiparetic Limbs in Stroke Patients before Intervention by Using Portable Stretching Device and EMG. J. Med. Biol. Eng..

[48] Chen J.J.J., Wu Y.N., Huang S.C., Lee H.M., Wang Y.L. (2005). The Use of a Portable Muscle Tone Measurement Device to Measure the Effects of Botulinum Toxin Type A on Elbow Flexor Spasticity. Arch. Phys. Med. Rehabil..

[49] Kumar R.T.S., Pandyan A.D., Sharma A.K. (2006). Biomechanical measurement of post-stroke spasticity. Age Ageing.

[50] Fazekas G., Horvath M., Toth A. (2006). A novel robot training system designed to supplement upper limb physiotherapy of patients with spastic hemiparesis. Int. J. Rehabil. Res..

[51] Pandyan A.D., Van Wijck F.M.J., Stark S., Vuadens P., Johnson G.R., Barnes M.P. (2006). The construct validity of a spasticity measurement device for clinical practice: An alternative to the Ashworth scales. Disabil. Rehabil..

[52] Nef T., Mihelj M., Riener R. (2007). ARMin: A robot for patient-cooperative arm therapy. Med. Biol. Eng. Comput..

[53] Takahashi C.D., Der-Yeghiaian L., Le V., Motiwala R.R., Cramer S.C. (2008). Robot-based hand motor therapy after stroke. Brain.

[54] Calota A., Feldman A.G., Levin M.F. (2008). Spasticity measurement based on tonic stretch reflex threshold in stroke using a portable device. Clin. Neurophysiol..

[55] Bovolenta F., Goldoni M., Clerici P., Agosti M., Franceschini M. (2009). Robot therapy for functional recovery of the upper limbs: A pilot study on patients after stroke. J. Rehabil. Med..

[56] Posteraro F., Mazzoleni S., Aliboni S., Cesqui B., Battaglia A., Dario P., Micera S. (2009). Robot-mediated therapy for paretic upper limb of chronic patients following neurological injury. J. Rehabil. Med..

[57] Posteraro F., Mazzoleni S., Aliboni S., Cesqui B., Battaglia A., Carrozza M., Dario P., Micera S. (2010). Upper limb spasticity reduction following active training: A robot-mediated study in patients with chronic hemiparesis. J. Rehabil. Med..

[58] Ferreira J., Moreira V., Machado J., Soares F. Biomedical device for spasticity quantification based on the velocity dependence of the Stretch Reflex threshold. Proceedings of the ETFA2011.

[59] Fazekas G., Zsiga K., Dénes Z. (2011). Robot-mediated upper limb physiotherapy: Review and recommendations for future clinical trials. Int. J. Rehabil. Res..

[60] Kim E.H., Jang M.C., Seo J.P., Jang S.H., Song J.C., Jo H.M. (2013). The Effect of a Hand-Stretching Device During the Management of Spasticity in Chronic Hemiparetic Stroke Patients. Ann. Rehabil. Med..

[61] Hu X., Tong K., Wei X., Rong W., Susanto E., Ho S. (2013). The effects of post-stroke upper-limb training with an electromyography (EMG)-driven hand robot. J. Electromyogr. Kinesiol..

[62] Ferreira J., Moreira V., Machado J., Soares F. Improved biomedical device for spasticity quantification. Proceedings of the 2013 IEEE 3rd Portuguese Meeting in Bioengineering (ENBENG).

[63] Sale P., Franceschini M., Mazzoleni S., Palma E., Agosti M., Posteraro F. (2014). Effects of upper limb robot-assisted therapy on motor recovery in subacute stroke patients. J. Neuroeng. Rehabil..

[64] Taveggia G., Borboni A., Salvi L., Mulé C., Fogliaresi S., Villafañe J.H., Casale R. (2016). Efficacy of robot-assisted rehabilitation for the functional recovery of the upper limb in post-stroke patients: A randomized controlled study. Eur. J. Phys. Rehabil. Med..

[65] Pennati G.V., Plantin J., Borg J., Lindberg P.G. (2016). Normative NeuroFlexor data for detection of spasticity after stroke: A cross-sectional study. J. Neuroeng. Rehabil..

[66] Dehem S., Gilliaux M., Lejeune T., Detrembleur C., Galinski D., Sapin J., Vanderwegen M., Stoquart G. (2017). Assessment of upper limb spasticity in stroke patients using the robotic device REAplan. J. Rehabil. Med..

[67] Lee D.J., Bae S.J., Jang S.H., Chang P.H. Design of a clinically relevant upper-limb exoskeleton robot for stroke patients with spasticity. Proceedings of the 2017 International Conference on Rehabilitation Robotics (ICORR).

[68] Calabrò R.S., Naro A., Russo M., Milardi D., Leo A., Filoni S., Trinchera A., Bramanti P. (2017). Is two better than one? Muscle vibration plus robotic rehabilitation to improve upper limb spasticity and function: A pilot randomized controlled trial. PLoS ONE.

[69] Posteraro F., Crea S., Mazzoleni S., Berteanu M., Ciobanu I., Vitiello N., Cempini M., Gervasio S., Mrachacz-Kersting N. (2018). Technologically-advanced assessment of upper-limb spasticity: A pilot study. Eur. J. Phys. Rehabil. Med..

[70] Sin M., Kim W.S., Cho K., Paik N.J. (2019). Isokinetic Robotic Device to Improve Test-Retest and Inter-Rater Reliability for Stretch Reflex Measurements in Stroke Patients with Spasticity. J. Vis. Exp..

[71] Htoon Z.L., Sidek S.N., Fatai S., Rashid M.M. Estimation of Upper Limb Impedance Parameters Using Recursive Least Square Estimator. Proceedings of the 2016 International Conference on Computer and Communication Engineering (ICCCE).

[72] Sidiropoulos A., Karayiannidis Y., Doulgeri Z. Human-robot collaborative object transfer using human motion prediction based on dynamic movement primitives. Proceedings of the 2019 18th European Control Conference (ECC).

[73] Oña E.D., Jardón A., Cuesta-Gómez A., Sánchez-Herrera-Baeza P., Cano-de-la Cuerda R., Balaguer C. (2020). Validity of a Fully-Immersive VR-Based Version of the Box and Blocks Test for Upper Limb Function Assessment in Parkinson’s Disease. Sensors.

[74] Scano A., Molteni F., Molinari Tosatti L. (2019). Low-Cost Tracking Systems Allow Fine Biomechanical Evaluation of Upper-Limb Daily-Life Gestures in Healthy People and Post-Stroke Patients. Sensors.

[75] Zhang L.Q., Chung S., Lin A., van Rey E., Bai Z., Grant T., Roth E. A portable intelligent stretching device for treating spasticity and contracture with outcome evaluation. Proceedings of the Second Joint 24th Annual Conference and the Annual Fall Meeting of the Biomedical Engineering Society, Engineering in Medicine and Biology.

[76] Ang W.S., Geyer H., Chen I.M., Ang W.T. (2018). Objective Assessment of Spasticity With a Method Based on a Human Upper Limb Model. IEEE Trans. Neural Syst. Rehabil. Eng..

[77] Jayaraman P.P., Forkan A.R.M., Morshed A., Haghighi P.D., Kang Y.B. (2020). Healthcare 4.0: A review of frontiers in digital health. Wiley Interdiscip. Rev. Data Min. Knowl. Discov..

[78] Heung H.L., Tang Z.Q., Shi X.Q., Tong K.Y., Li Z. (2020). Soft Rehabilitation Actuator With Integrated Post-stroke Finger Spasticity Evaluation. Front. Bioeng. Biotechnol..

[79] Oña E.D., Garcia-Haro J.M., Jardón A., Balaguer C. (2019). Robotics in health care: Perspectives of robot-aided interventions in clinical practice for rehabilitation of upper limbs. Appl. Sci..

[80] Yee J., Low C.Y., Ong P., Soh W.S., Hanapiah F.A., Zakaria N.A.C., Enzberg S.v., Asmar L., Dumitrescu R. (2020). Verification of Mathematical Model for Upper Limb Spasticity with Clinical Data. Iop Conf. Ser. Mater. Sci. Eng..

[81] Fleuren J.F., Voerman G.E., Erren-Wolters C.V., Snoek G.J., Rietman J.S., Hermens H.J., Nene A.V. (2010). Stop using the Ashworth Scale for the assessment of spasticity. J. Neurol. Neurosurg. Psychiatry.

[82] Stein J., Krebs H.I., Frontera W.R., Fasoli S.E., Hughes R., Hogan N. (2004). Comparison of Two Techniques of Robot-Aided Upper Limb Exercise Training After Stroke. Am. J. Phys. Med. Rehabil..

[83] Hermens H.J., Freriks B., Disselhorst-Klug C., Rau G. (2000). Development of recommendations for SEMG sensors and sensor placement procedures. J. Electromyogr. Kinesiol..

[84] Riener R., Nef T., Colombo G. (2005). Robot-aided neurorehabilitation of the upper extremities. Med. Biol. Eng. Comput..

[85] Toigo M., Flück M., Riener R., Klamroth-Marganska V. (2017). Robot-assisted assessment of muscle strength. J. Neuroeng. Rehabil..

[86] Hu X.L., Tong K.Y., Song R., Zheng X.J., Lui K.H. Robot-assisted wrist training for chronic stroke: A comparison between electromyography (EMG) driven robot and passive motion. Proceedings of the 2008 2nd IEEE RAS & EMBS International Conference on Biomedical Robotics and Biomechatronics.

[87] Malhotra S., Cousins E., Ward A., Day C., Jones P., Roffe C., Pandyan A. (2008). An investigation into the agreement between clinical, biomechanical and neurophysiological measures of spasticity. Clin. Rehabil..

[88] Jobin A., Levin M.F. (2000). Regulation of stretch reflex threshold in elbow flexors in children with cerebral palsy: A new measure of spasticity. Dev. Med. Child Neurol..

